# Image analysis tools for improved characterization of nuclear chromatin patterns by confocal fluorescence microscopy

**DOI:** 10.1007/s00249-025-01770-y

**Published:** 2025-06-23

**Authors:** Mohammadmehdi Roushenas, Marco Salerno, Virginia Bazzurro, Elena Gatta, Alberto Diaspro

**Affiliations:** 1 Dipartimento Di Fisica, Università Di Genova, Genoa, Italy; 2 Nanoscopy and NIC@Iit, CHT, Istituto Italiano Di Tecnologia, Genoa, Italy

**Keywords:** Cell nuclei, Chromatin compaction, Confocal microscopy, Hoechst, Fractal dimension

## Abstract

We have collected fluorescence images of fixed cell nuclei of two different types—HeLa and HepG2—with DNA labeled by a standard fluorophore, and have devised three different quantitative parameters aimed to describe the distribution of the nuclear chromatin. The parameters are the fractal dimension, associated with the intricacy and hierarchical structure of chromatin; the total perimeter of local maxima, associated with the amount of chromatin domains; and the radial distance of angularly averaged intensity profile maximum, associated with the possible occurrence of a peak density at a characteristic distance from the nucleus center. Our results suggested that it was possible to differentiate the two types of cells in the 3D space of the defined parameters. Therefore, these parameters appear promising in identifying specific functional patterns in chromatin. At the same time, the negative control of different runs of measurements on the same cell type also showed at least partial differentiation. Thus, the tool proposed here for nuclear chromatin pattern characterization is probably sensitive to the cell life cycle moment almost as much as to the cell type and should be tested further on cells synchronized at the same phase during their cycle.

**Supplementary Information:**

The online version contains supplementary material available at 10.1007/s00249-025-01770-y.

## Introduction

Pointing to the importance of chromatin is an easy task, supported by such a wide variety of literature background (Van Steensel 2011) (Mishra et al. 2023). In short, chromatin is a complex of nucleic acids and proteins responsible for packing genetic information in living cells and for its transfer to daughter cells. To this goal, the extraordinarily long DNA chain in humans (almost two meters) is packed in the 10 µm-scale size of the cell nucleus in such a way that it is still ready to be unzipped and accessed for replication at the right moment (Kak 2023). The chromatin structure presents multiple organization levels, spanning from micrometer down to nanometer scale (Mirny 2011), from the single DNA chain size, wrapped around histones octamers to form nucleosome spools, to the bead-on-a-string chromatin fiber in the scale of 10 nm. Nowadays, even the lower levels are accessible thanks to super-resolution microscopy (Kostiuk et al. 2019) (Benke and Manley 2012) (Burgers and Vlijm 2023) (Lakadamyali and Cosma 2015)(Burgers and Vlijm 2023) (Pierzynska-Mach, Czada, et al., 2023b), which can primarily be used for addressing specific interactions occurring in live cells during transcription, translation and even repair of DNA. Therefore, the relevance of a study based on imaging with diffraction-limited resolution (as large as at least 200 nm) by simple confocal microscopy could be questioned. However, the size scale of the chromatin structure is already known, mainly after cryo-electron microscopy studies (Takizawa and Kurumizaka 2022), and the closest higher level has also been clarified by small-angle X-ray scattering (SAXS) studies (Nishino et al. 2012). For example, more than one decade ago, the latter technique made it possible to clarify about the hypothesized existence of a higher level of chromatin fibers in the size scale of 30 nm, concluding that there is not necessarily such a regular structure in human mitotic chromosomes (Hansen 2012). Nishino et al. also investigated the size range of 50–1000 nm by ultra-SAXS, showing that no regular structures are present in human mitotic chromosomes even on that scale (Nishino et al. 2012). On the other hand, with chromatin not really being regular, yet somewhat hierarchically arranged in an efficient way to allow for its high level of compaction, over the years several researchers have put forward the hypothesis of a fractal arrangement extending in the submicrometric size scale. In this intermediate mesoscale size, chromatin structure analysis falls under the domain of confocal microscopy. Therefore, we have tried to use this imaging technique to investigate chromatin organization, as we think that, even on such a relatively large scale across the micrometer, chromatin organization still has to disclose helpful information. This has already been observed in the past (Mascetti et al. 1996), yet should be reconsidered with current image analysis capabilities (see, e.g., Pierzynska-Mach, Cainero, et al. 2023a). Indeed, in this work, one of the parameters extracted from our images is the fractal dimension (FD). However, we have worked out two more parameters, trying to express both the level of chromatin density and its distribution with the distance from the nucleus center, namely the total perimeter of domains (TPD) of chromatin and the radial position of maximum density, R_max_, respectively. The goal was to find a set of descriptors that will allow the distinction of chromatin arrangement between different cell types and, in perspective, also between healthy and diseased cell conditions.

The high complexity of the cell nucleus (Maraldi et al., 2003) goes along with chromatin organization. Investigations of chromatin distribution patterns started already several decades ago, by simple methods such as evaluation of optical density, which disclosed differences between cell types or functioning conditions (Durie et al. 1978; Sahota et al. 1986). More recently, changes in chromatin patterns of neurons were associated with developmental disorders (García-Cabezas et al. 2018). Our preliminary work was not intended to provide insights into the functional properties of this organization. Rather, we aim to suggest a tool for future studies correlating functional characterization with the structural distribution of chromatin, as observable in conditions not too far from native ones, by optical fluorescence microscopy.

## Experimental

### Sample preparation

Both cell types, namely HeLa and human hepatocellular carcinoma (HepG2) cell line, were cultured in 90% Dulbecco’s modified Eagle’s medium–high glucose (DMEM, Sigma-Aldrich, St. Louis, MO, USA, product No. D5796), 10% fetal bovine serum (Sigma-Aldrich, St. Louis, MO, USA, product No. F9665), 2.0 mM glutamine (Sigma-Aldrich, St. Louis, MO, USA, product No. G7513), and 100 IU/mL penicillin–streptomycin (Sigma-Aldrich, St. Louis, MO, USA, product No. P4333) and maintained at 37 °C in a humidified atmosphere of 95% air and 5% CO_2_. The passage number of the cell cultures was eight for HeLa (P8) and seven for HepG2 (P7). The cells were plated at a density of 5.0–10 × 10^4^ cells on 18 mm diameter poly-L-lysine-coated glass coverslips (Sigma-Aldrich, St. Louis, MO, USA, product No. P2636). For the labeling, the cells were fixed with formaldehyde solution 4%, buffered, pH 6.9 (Sigma-Aldrich, St. Louis, MO, USA, product No. 1.00496) for 15 min at room temperature. After washing twice with Dulbecco’s phosphate-buffered saline (DPBS, Sigma-Aldrich, St. Louis, MO, USA, product No. D8662), the cells were stained with 2.5 µM Hoechst (Sigma-Aldrich, St. Louis, MO, USA, product No. 14533) at room temperature for 30 min. Following three washes of 5 min each with DPBS, the coverslip was mounted on a microscope glass slide (1″ × 3″) with ProLong™ Glass Antifade (Thermo Fisher Scientific, product No. P36980).

### Fluorescence microscopy imaging of cell nuclei

**Fig. 1:**
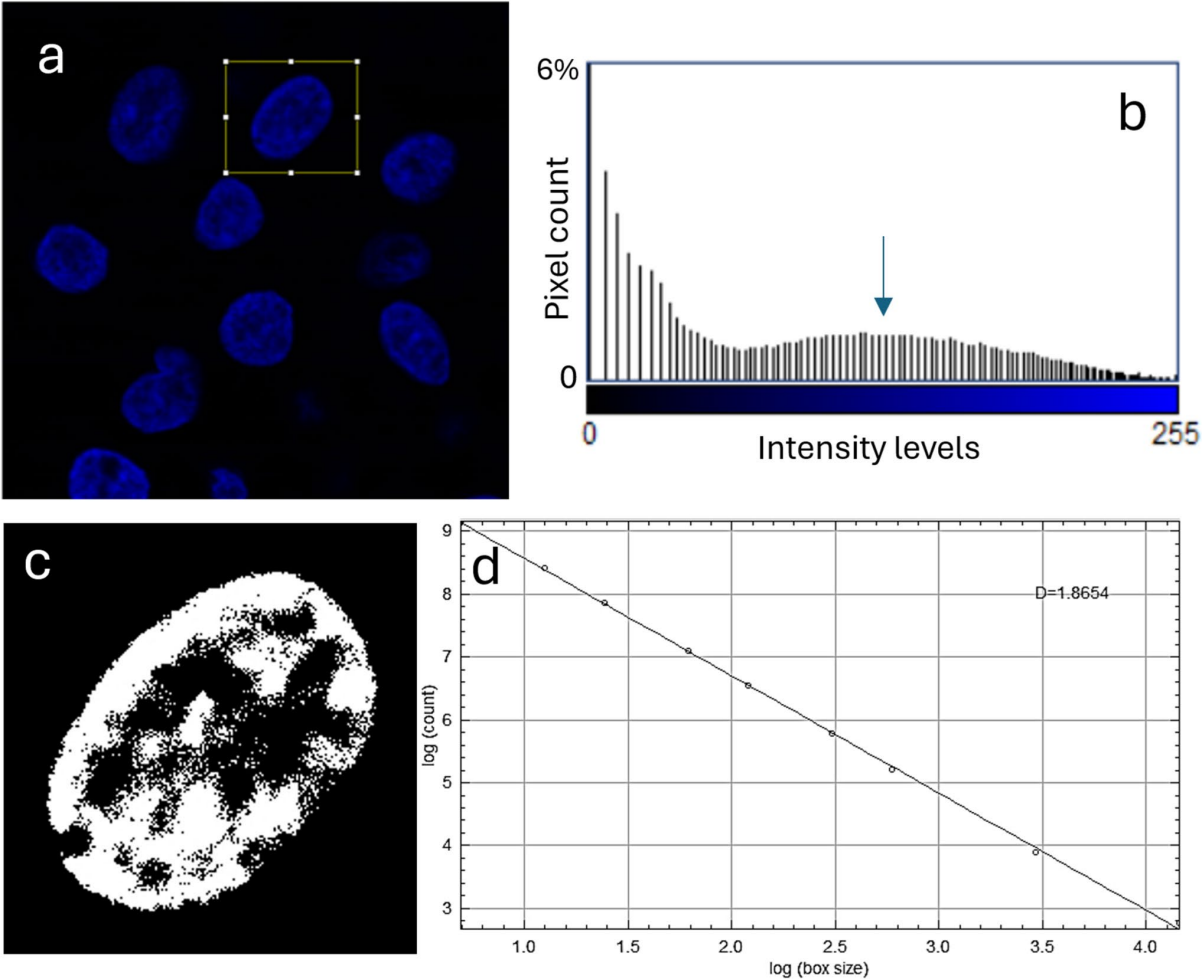
Extraction of FD value. **a** Original image, with selection of ROI to be cropped. **b** Histogram of intensity levels obtained after equalization, used to find the threshold for binarization (see arrow). **c** Resulting binarized image. **d** Log–log plot of intensity versus box size, the slope is the FD

We used a confocal inverted microscope Stellaris 8 by Leica Microsystems (Mannheim, Germany), utilizing the doughnut-shaped laser for stimulated emission depletion (STED), with 775 nm wavelength, at maximum available intensity (100% power), for two-photon excitation (2PE) of Hoechst as the nuclei labeling fluorophore (see Discussion for more detailed considerations). For each sample, we collected several images (8-bit intensity scale) of different regions, each containing, in turn, several different nuclei (see Fig. 1a). The expected resolution in confocal mode was diffraction limited, i.e., around 250 nm in the xy focal image plane. Therefore, according to the Nyquist–Shannon rule (Diaspro and van Zandvoort 2021), we set a pixel size of 100 nm, a little less than half the expected resolution, which allowed us to cover several nuclei with a typical image digital size of 512 × 512 pixels.

### Image analysis

The images, saved with the proprietary Leica software LasX, were then processed with Fiji distribution of ImageJ (Abràmoff et al. 2004) according to the protocols described next for each of the three parameters of interest to be extracted. The first step for all parameters was cropping the image (command *Image*-*Crop*) to isolate the single nucleus of interest; see, e.g., the rectangular region of interest (ROI, yellow edges) in Fig. 1a.

#### Fractal dimension, FD

The FD of the patterns inside the images of nuclei was obtained with a dedicated Fiji plugin, based on the common method known as “box counting” (Russ 1994, 2011). After cropping, we first equalized the distribution of intensity levels (*Process*-*Enhance contrast*, *Equalize histogram*, with Saturated pixels set to 0.35%), such that its histogram appeared to be similar to that shown in Fig. 1b. Then, we binarized the image of the single-nucleus setting the manual threshold (*Image*-*Adjust*-*Threshold*) to the peak position of the intensity levels associated with the fluorophore (center of the broadband on the right side, see arrow in Fig. 1b). The result of this binarization was typically as shown in Fig. 1c. On this image, we applied command *Analyze*-*Tools*-*Fractal box count*, setting the Black background option. The result is a log–log plot, as shown in Fig. 1d, whose slope is the FD value.

#### Total perimeter of chromatin domains, TPD

**Fig. 2:**
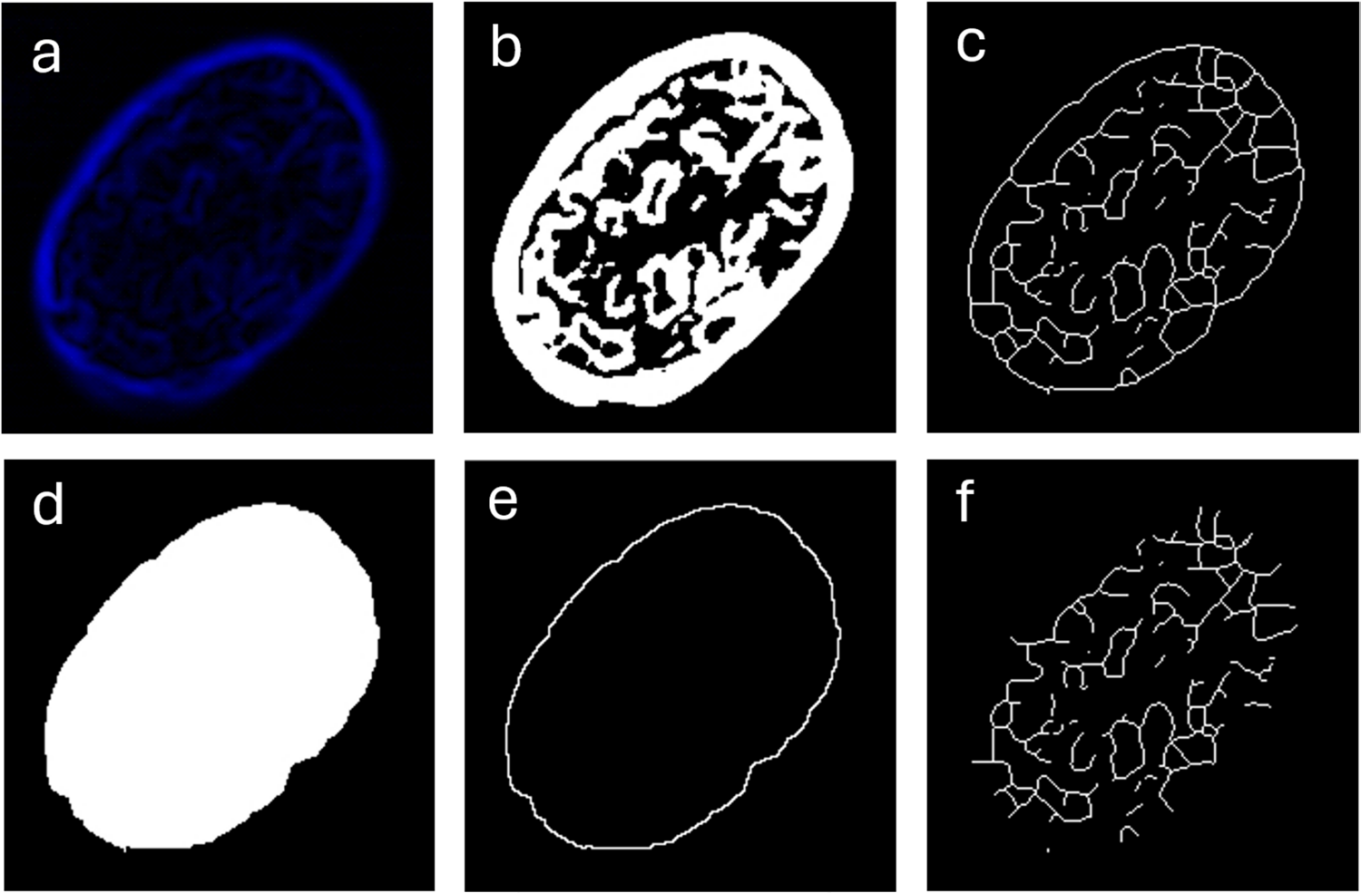
Extraction of the TPD value. **a** Result of Sobel edge detection filter. **b** Same image as in (**a**), after binarization. **c** Result of skeletonization. **d** Same image as in (**b**) after filling holes operation. **e** Result of erosion of (**d**) by 1 pixel and outline detection. **f** Result of subtraction (**c**)-(**e**); the TPD is calculated after the total length of these white lines, divided by the nucleus area in (**d**) (for normalization purposes)

In Fig. 2, the protocol used to extract parameter TPD from the nuclei images is shown. After cropping and equalizing the single-nucleus image, as in Fig. 1a, b, the image was first low-pass filtered by *Process*-*filters*-*Gaussian blur* with sigma radius 2 to remove possible noisy pixels. Then the edges of the high-intensity domains were identified by *Process*-*find edges*, which implements a Sobel filter for edge detection (basically, 2D derivative along major image axis x and y), see Fig. 2a (Russ 2011). Then, the image of edges was thresholded by *Image*-*adjust*-*threshold*, B&W option with dark background, setting the top range cursor in the middle (highest point) of the right band in the histogram. The so-binarized edges of the chromatin domains are shown in Fig. 2b. This image was then skeletonized by *Process*-*binary*-*skeletonize* (Fig. 2c). The whole nucleus area was obtained next by applying to the former stage image (Fig. 2b) the command *Process*-*binary*-*fill holes* (See Fig. 2d). The final goal was to isolate the nucleus outer edge, to be able to remove it from the inner chromatin domain edges. To this aim, the whole white image of the nucleus was eroded one pixel in width, by *Process-binary-erode*, and the outline was obtained by *Process-binary-outline*. Next, we used the *Process*-*image calculator* to subtract the last obtained outline (Fig. 2e) from the previous skeletonized image (Fig. 2c). We then went back to the filled nucleus image (Fig. 2d) and selected *Analyze*-*Set measurements*, *Area fraction*. With *Analyze*-*Measure*, the % Area of the rectangular image corresponding to the inner nucleus region was obtained and annotated. On the skeletonized image, again we applied *Analyze-Measure* and obtained the total length of chromatin domains. The TPD value was finally obtained by normalizing the latter value to the previous one obtained for the nucleus area, such as to remove the effect of nucleus size.

#### Radial position of maximum intensity, R_max_.

**Fig. 3:**
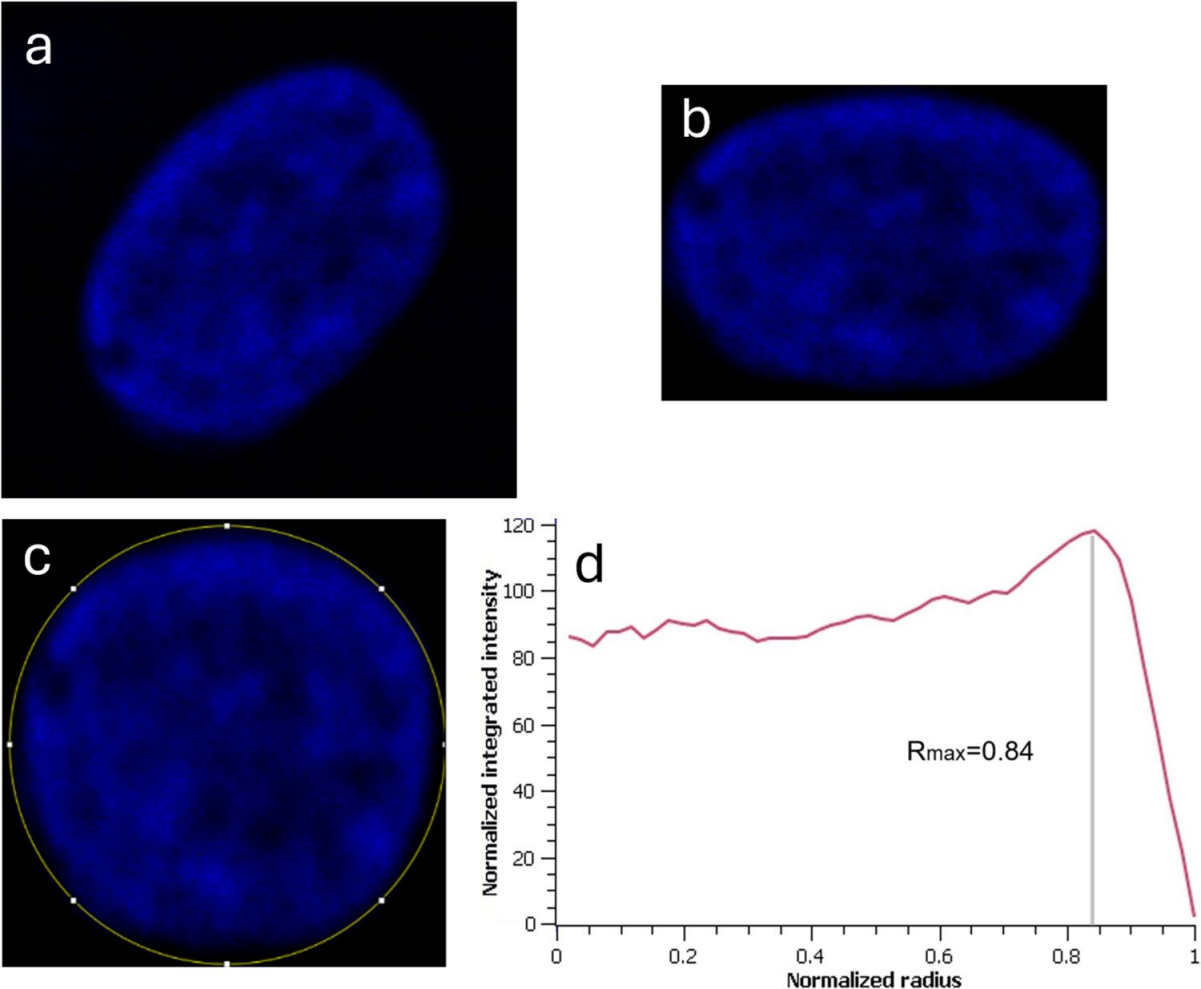
Extraction of R_max_ value. **a** Typical image of a single nucleus after cropping. **b** The same nucleus as in (**a**) after rotation to align the main axis of the ellipsoidal nucleus to the horizontal and vertical axis of the image, and cropping further close to the nucleus edges (1 pixel background only). **c** Image after stretching the short side in (**b**) to the same size as the long side to make it square (nucleus circularization). **d** Mean radial profile averaged across all directions at 360° (step 1°) around the center of the circle in (**c**), with renormalization of circle radius to 1

In Fig. 3, the protocol used to extract parameter R_max_ from the nuclei images is shown. In particular, to determine this parameter, we discarded nuclei with clearly asymmetric shape (protrusions or dents), and considered only nuclei with roughly elliptical shape. Examples of the shapes of discarded nuclei have been shown in Fig.S3. After cropping and equalizing the single-nucleus image (Fig. 3a), the main axis of the—approximately—elliptical nucleus was aligned with the rectangular image axis by rotation, with *Edit*-*Selection*-*Rotate*. The following step consisted of a new cropping operation, aiming to select just the rectangle containing the nucleus and almost touching its edges; only 1 pixel outside the edge was maintained for the sake of avoiding any possible loss of information (see Fig. 3b). Then, we performed what we called a “circularization” of the nucleus, meaning that the shorter size of the rectangular image was stretched until it reached the same pixel size as the long one, by *Image*-*Adjust*-*Size*, (Fig. 3c; the “Keep aspect ratio” option was de-selected during this operation). Next, we applied the Fiji plugin Radial_class (Baggethun 2002). All the 360 profiles starting from the center of the image—and circularized nucleus—and pointing around each at 1° angular spacing were so averaged to provide a single profile (Fig. 3d). To compare the profiles from nuclei of different sizes, the x-axis in the plots (radial distance) was normalized for each profile to its maximum value, such that it then appeared to span the range from 0 to 1. At this point, we sought the x position at which the absolute maximum in the profile occurred, which was called R_max_.

### Plotting and statistical analysis

For the basic 1D graphic profiles, SciDAVis 2.7 was used (*SciDAVis Plotting Software*, n.d.). For 3D scattering plots in the space of chromatin describing parameters, we used SigmaPlot 12.0 (Grafiti, Palo Alto, CA, USA, (*SigmaPlot Web Page*, n.d.)). This software was also used for comparing the populations with one-way ANOVA; the Tukey’s pair test was carried out, looking at different statistically significant levels for the differences, i.e., *p* < 0.05 (identified with a single star symbol, *), *p* < 0.01 (two stars, **), and *p* < 0.001 (three stars, ***). For the 3D rendering with ellipsoidal volumes (Fig.S4), MATLAB R2023b was used (The MathWorks, Inc., Natick, MA, USA, (*MATLAB Web Page* 2023)).

## Results

**Fig. 4:**
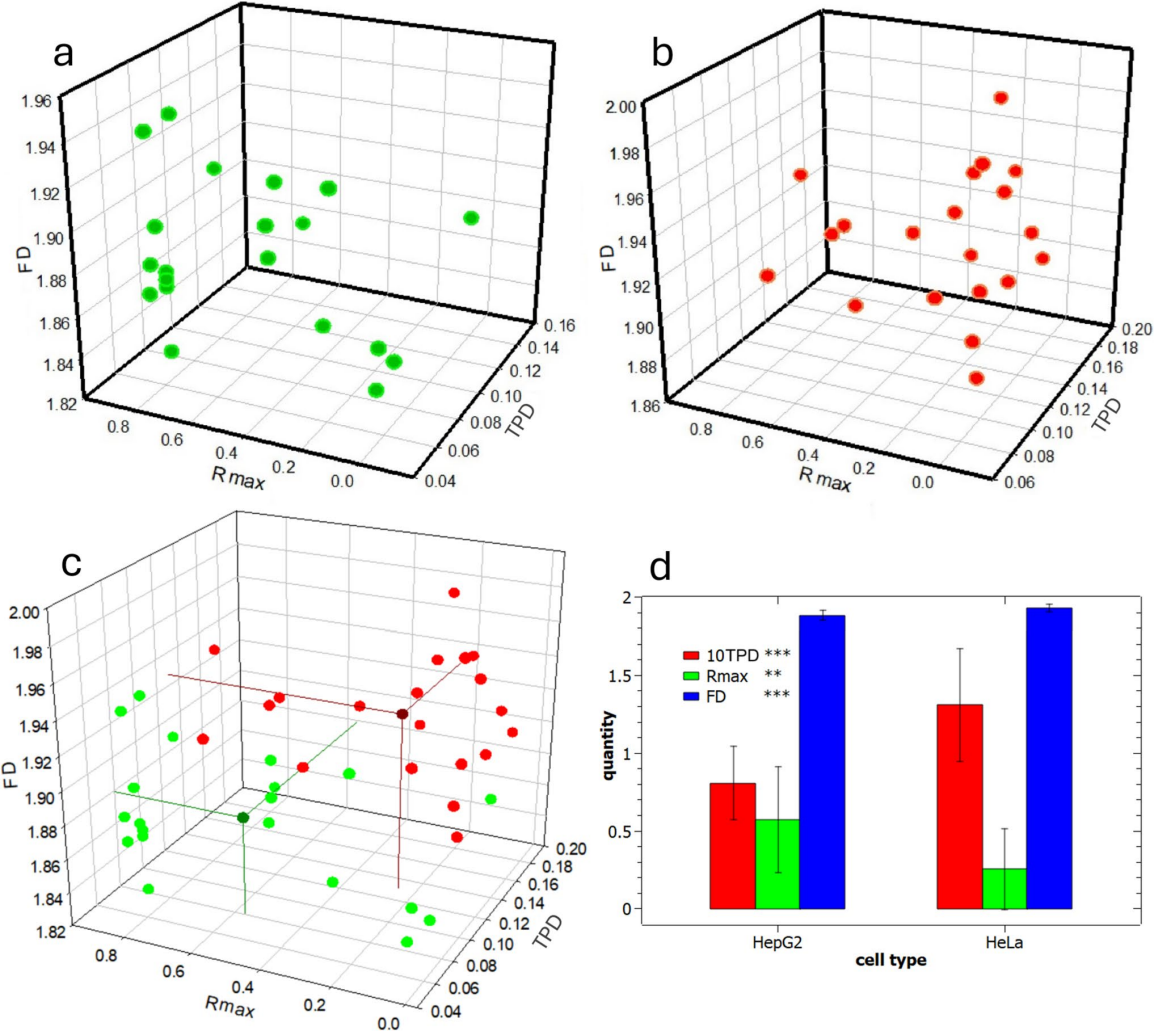
Experimental data for comparison of HeLa and HepG2 cell nuclei chromatin patterns during run#1 experiment. **a** Data points for HeLa cells, **b** data points for HepG2 cells, **c** combined data of (**a**) and (**b**) with the respective mean points, **d** plot of means ± standard deviation for each of the three parameters for the two cell types (TPD in shown red, R_max_ in green, and FD in blue); results of ANOVA showed significant difference in all cases

In Fig. 4a, the data resulting from the experiment called run#1 are reported to compare the two cell types, HeLa and HepG2. The images of the nuclei used to extract those data have been included as Fig.S1,2 for each cell type, separately. Clearly, on inspection of those images from a first sight by the naked eye, no significant difference emerges between the two populations, given the intra-population variability. Nevertheless, a deeper insight is expected based on the distribution of values for our three selected parameters.

In Fig. 4a, b, the 20 data points of each of the HeLa and HepG2 cell nuclei in the 3D space of the parameters are shown, respectively. In Fig. 4c, both are shown again together, each with the additional mean data point, in the same respective color—namely green for HeLa and red for HepG2—in a darker shade to be recognized. For the mean points, the projections to the three embedding planes of the parameters in the space have also been traced, to allow for better identification of the 3D position. Additional alternative representations of these data in the 3D space are shown in Fig.S4, which allows to visualize the overall positions of the respective populations as ellipsoidal clusters. Finally, in Fig. 4d, the same mean values of 3D coordinates are plotted for each cell type, with error bars representing ± 1 standard deviation. For all three parameters, statistically significant differences appeared between the populations of the two cell types. The difference between the mean values of FD seems to be very low; nevertheless, the error bars are also much smaller for this parameter than for the other two—probably due to a more robust measurement procedure or intrinsically characteristic nature of the FD quantity itself. As a result, the means are statistically different, with very high significance level (***). The same marked statistical difference appears for TPD (***). Finally, for R_max_, where apparently some overlap of the respective distributions emerges, the means are still significantly different, even if with a lower significance level (**).

One critical point was to evaluate if the observed differences were simply due to the random occurrence of other factors not associated with the intrinsic differences between the nuclei populations. Therefore, as a first step, the experiment was repeated, which provided the dataset called run#2. These results are shown in Fig.S5 and basically confirm the differences between the two populations of cells. Not only were the pairs of values for each parameter in Fig.S5 different, as previously observed in Fig. 4, but—above all—the observed differences pointed to the same direction. Actually, also in run#2, HeLa nuclei showed higher TPD (***), lower R_max_ (*), and higher FD (***) than HepG2 nuclei, with only a minor decrease in overall difference scores (with a total score of seven “star” units instead of eight).

## Discussion

One technical limitation during our measurements was that the Hoechst fluorophore is usually excited—by single photons—in the UV range of the light spectrum and undergoes consequent emission in the blue region. However, our confocal microscope has no UV excitation source; instead, it has a supercontinuum white light laser in the 440–800 nm range. Initially, we also tried to excite Hoechst in the long wavelength foot of its absorption spectrum at 440 nm, but the signal was of too low quality (SNR ≈1). Therefore, we used the other laser available in the system for stimulated emission depletion, working at 775 nm wavelength, in two-photon excitation mode (2PE) (Diaspro et al. 2007). This way, two photons impinging simultaneously on the sample (on the timescale of the order of 1 fs) can contribute each roughly half the energy—and thus have twice the wavelength—than the single-photon excitation case. Of course, much higher peak laser power is required for 2PE (we used it at 100% power, corresponding to ≈400 mW at the focal plane), which obviously is still not too invasive, thanks to the point of the laser being pulsed (at 80 MHz). Therefore, our source used in 2PE mode was equivalent to single-photon excitation with a wavelength of ≈388 nm, well within the Hoechst absorption spectrum. Whereas using a doughnut-shaped beam is expected to somewhat diminish the confocal resolution expected after the theoretical diffraction limit, it has been shown in (Bianchini and Diaspro 2012) that in practice only a minor effect is observed in this direction, when a good signal-to-noise ratio is obtained.

Since our confocal images were diffraction-limited to ≈250 nm resolution, according to the Nyquist–Shannon sampling rule we selected a pixel size ≈100 nm. This relatively large pixel size, associated with an image size of 1024 × 1024 pixels, i.e.,102.4 × 102.4 μm^2^, allowed us to image several nuclei within a single image scan. Thus, we had quite some redundancy in the number of nuclei to select for analysis in our experiment. We decided to select the nuclei that showed best contrast and were neither affected by protruding bumps or intruding cavities, nor too much elongated or deviating significantly from oval shape (see Fig.S3). The last requirement was due to the point of having to approximate the nuclear envelope with an ellipse, see"Image analysis". As mentioned in the Experimental section, we found it convenient to acquire images with a field of view sufficiently large to collect several nuclei per image instead of a single one. This is because the typical size of a single nucleus is relatively small (maximum around 10 µm diameter), and at the set digital resolution (around 100 nm per pixel), scanning single-nucleus images would require little more than 100 × 100 pixels, which would have definitely been unnecessarily small. This allowed us to work pretty fast in the imaging step. Most of the work done in this experiment actually focused on defining the protocols for calculating the parameters, applying them to our images, and analyzing the statistical significance of our results.

The importance of FD in describing chromatin structure has already been pointed out extensively in the literature (Almassalha et al. 2017; Boettiger et al. 2016; Metze et al. 2019; Mirny 2011; Yi et al. 2015). There exist several other methods for carrying out the fractal analysis of images in Fiji, according to different plugins (e.g., Fraclac (Karperien 2012), which can also calculate lacunarity (da Fonseca de Albuquerque et al. 2022; Ţălu et al. 2020)). However, we decided to keep extraction of this traditional image parameter as simple and straightforward as possible, using the default FD calculation method of Fiji. Binarization for subsequent extraction of FD is a somewhat arbitrary step in that the result depends critically on the position set for the threshold on the signal intensity of DNA inside the nucleus. We initially tried several automated criteria available in Fiji for this binarization operation (the better ones apparently being, in our case, Moments or Otsu). However, we did not find consistent results throughout all the nuclei images when doing so. Therefore, we preferred to set the threshold manually. Since we did not subtract the background offset and set the threshold considering all the pixels in the cropped nucleus image (rectangular area including background), the narrow population of lowest background counts appeared in the distribution of intensity levels, which was clearly distinguishable from the population of bright pixels corresponding to labeled nucleus regions, recognizable as a much broader symmetric band centered around higher intensity levels. In all cases, we set our threshold to the peak position of this band (corresponding also to the median value).

The parameter called here TPD has already been used in Irianto et al. ((Irianto et al. 2014)), where it was used to describe the change in chromatin pattern of chondrocytes on altering their osmotic pressure. They called this quantity with the different name of CCP, namely chromatin condensation parameter. We preferred not to adopt the same name, as we think that this parameter, while useful, is not by itself alone a full descriptor of the chromatin condensation. Actually, the two parameters of CCP and FD together describe to deeper extent the chromatin compaction, in terms of intensity and characteristic sub-structure spacing, respectively.

The third parameter R_max_ was selected to identify the characteristic distance from the nucleus center at which the maximum chromatin compaction, described by FD and TCP, would eventually occur. One possible criticism about R_max_ points to the nonlinear change applied to the nuclei center-to-edge distances during circularization. However, the advantage of making it possible to compare all radial distances and—after normalization to 1—for nuclei of all size, makes this parameter interesting. In perspective, R_max_ could eventually be associated with the amount of balance between euchromatin, which is assumed to stay closer to the nuclear lamina, and heterochromatin, which is supposed to remain closer to the inner regions of the nuclei. However, for the moment this is just bare speculation, as we have already pointed out that insight in functional interpretation of the observed differences is beyond the scope of the present work. So, we limit our comments to the performance of our three parameters in describing the differences between the two populations of test cell nuclei studied here.

In calculating R_max,_ we made a selection of nuclei of elliptical shape only, discarding the irregularly shaped ones (for details see Experimental section and Fig.S3). The discarded nuclei were actually a small part of the total, around 30%, for both cell types. Additionally, whereas we are aware that nuclei can have irregular morphology due to a number of factors, both physiological and pathological, as well as an effect of sample preparation, there are no reasons to doubt that also the subset of regular (i.e., elliptical)-shaped nuclei is anyway representative of the whole population of the respective cell type.

**Fig. 5:**
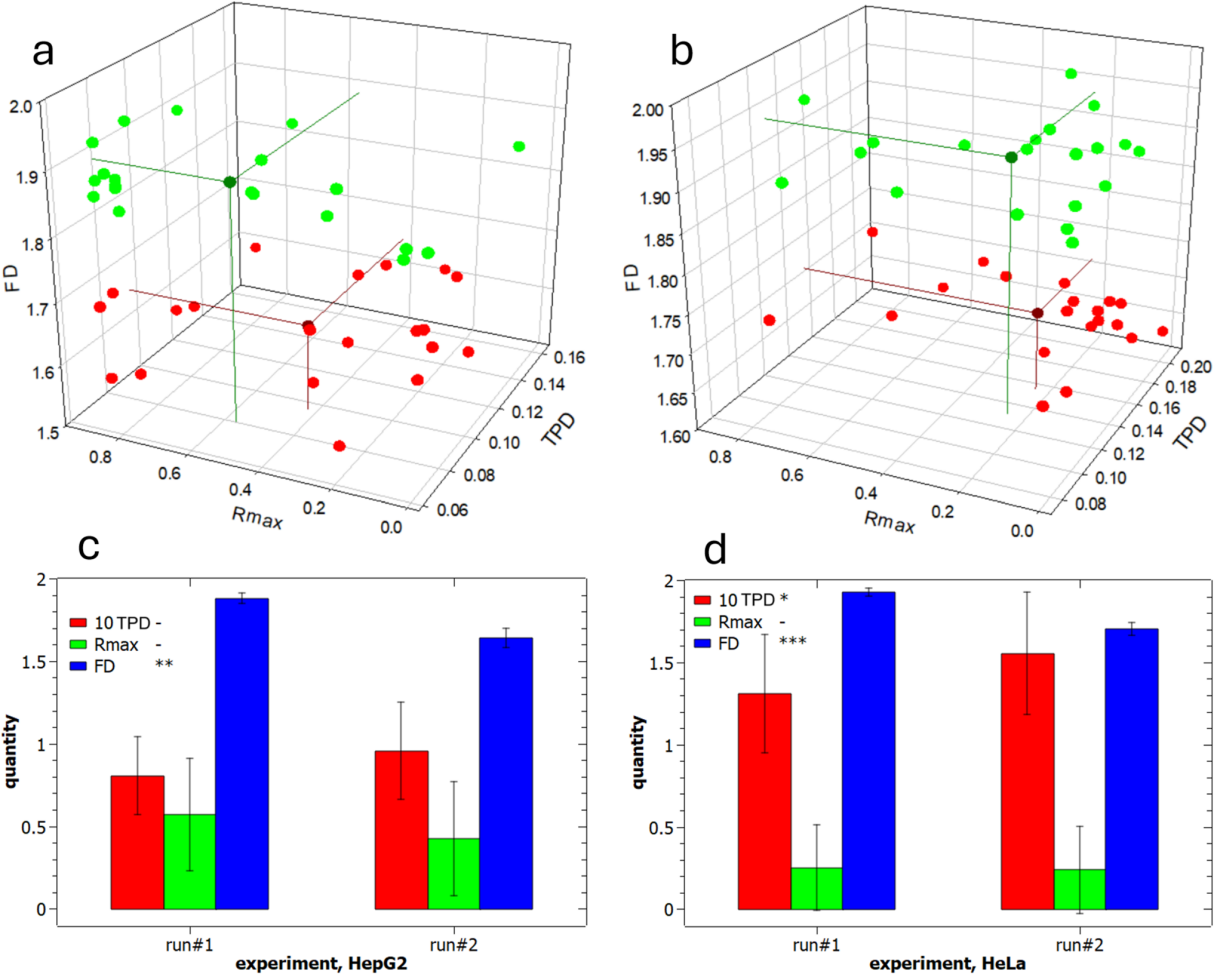
Experimental data for comparison of run#1 and #2 datasets. **a** Run#1 and run#2 data points for HepG2 cell nuclei, **b** run#1 and run#2 data points for HeLa cell nuclei. **c** and **d** present the respective plots (for HepG2 and HeLa nuclei) of means ± standard deviation for each of the three parameters for the two runs of the experiment (TPD in shown red, R_max_ in green, and FD in blue); results of ANOVA showed significant differences in several cases (for details see main text)

The significance of the differences between the means of all R_max_ between HepG2 and HeLa cell nuclei was assessed with the ANOVA test shown in Figs.4d and Fig. 5 (and confirmed with the same in Fig.S5d). However, we can also wonder if the means of all radial profiles for a single nuclei population can give similar insight, and how the positions of the maximum for the mean profile compares with the mean positions of the maxima for each individual profile. Therefore, we carried out a similar analysis for the dataset of experiment run#1, the same represented in Fig. 4, and the results are shown in Fig.S6 (details see Supplementary Information). Obviously, the position of the maximum of the mean plots does not coincide with the mean of the positions of each maximum, as the process of extracting a maximum is nonlinear. Nevertheless, qualitatively the information obtained is similar: we see from Fig.S6 that the HepG2 nuclei exhibit a maximum of the mean profile shifted quite close to the outer edge, at ~ 0.85, whereas for the HeLa nuclei this maximum occurs at ~ 0.13. These positions are clearly more spaced apart from each other than those resulting from the means (0.57 and 0.25, respectively), yet they rank in the same order. In Fig.S6, the bands around the mean profile in both cases show the observed uncertainty: in red, the band associated with ± 1 standard deviation width is shown, whereas in green is the band with ± 1 standard error. In both cases, despite the relatively large uncertainty, the different behavior is clear and confirms and strengthens the difference observed based on the population of single R_max_ values.

Overall, Fig. 4 (run#1) and Fig.S5 (run#2) show significant differences between the cell types considered. However, despite the results being confirmed during the second, repeated experiment, these could still be due to a lucky and unlikely occurrence of, e.g., systematic errors and possess no real physical meaning. Therefore, the final cross-check requested to validate our observations would be that the two datasets, run#1 and run#2, do not show statistically significant differences—at least not to the same degree—between the old (run#1) and new (run#2) set of data for the same cell type. To test this null hypothesis, we carried out a similar comparison as that between the cell types, and the results are presented in Fig. 5. Actually, from the panels in Fig. 5c, d one can see that the ANOVA pointed out some statistically significant differences also in these two comparisons. In particular, FD differed between run#1 and #2, both for the HepG2 (**) and—even more—for the HeLa nuclei (***). However, for the other two parameters, only in one case out of four a significant difference appeared, for the TPD values of HeLa nuclei, which was at the lowest significance level (*). HeLa cells appear to be more variable across their own total population (a total of four "stars" difference for all parameters vs the two "stars" of HepG2). In particular, FD is the most sensitive parameter (2 "stars" on average): it may be the case that the evaluated uncertainty for this figure is somewhat underestimated. We conclude that its apparent robustness is more from the point of view of mathematical calculation rather than in the physical contents. Nevertheless, overall, a total—average—score of 3 "stars" emerged (2 for HepG2 and 4 for HeLa), as compared to the average score in the difference of 7.5 "stars" between cell types (8 for run#1 and 7 for run#2). Therefore, this much lower extent of observed differences at least partly confirms our hypothesis in support of using the selected parameters. The minor differences between different sampling datasets for the same cell type may be due to a relevant intrinsic variability of each cell population, following the point that the cells are not synchronized in a given phase of their life cycle, as well as to the limited statistics, an effect that could be mitigated in the future by taking a larger number of images for each case, e.g., *N* = 40 or 60.

Another limitation of our work is in the actual biological scope. In fact, the selection of the cell lines did not correspond to any particular biological question, nor did we intend to draw any conclusions about the compared cell lines. We are aware that, for example, even the prolonged passaging of cell cultures may affect nuclear morphology and chromatin compaction, and this effect should be taken into account when comparing cells with a precise biological goal. However, the present types of cells have been selected only as representative of different cell populations, as test samples to see if any difference may be found by our set of describing parameters, and how strong this discrimination can be.

In view of possible future refinement of the image analysis protocol presented in this work, averaging the radial profiles of nucleus intensity (here aimed at obtaining the R_max_ values) appears to be the procedure with the most potential for additional insight. As shown previously, the results occurring after averaging R_max_ values in a population can be cross-checked by looking at the single R_max_ from the mean profile obtained after averaging them altogether (as in Fig.S6). Additionally, the absolute maximum is probably just a piece of information hidden in the respective profile. Complementary data could be the prominence of such a maximum—how much above the mean intensity—and the possible occurrence of a minimum (other than the zero close to normalized radial distance *x* = 1) or the presence of a secondary maximum in a different position.

## Conclusion

In this work, three parameters were defined to describe the different patterns in chromatin distribution inside cell nuclei. These parameters were tested on fixed cell samples of cell types available at our laboratory, representing two types of cancer cells, as test samples. Altogether, the identified 3D space of parameters appeared to allow successful distinction of the cell types. Some variations between different subsets of the same type of cells also appeared. This is probably due to the dependency on the cell life cycle, which adds to the different types of tissue cells. Nevertheless, we have here laid the foundations for a methodological approach to cell nuclei image analysis, which could be used in the future for more comprehensive studies, including improved control of cell samples, for example, including controls of healthy cells with the same type of tissue as the sick ones (e.g., healthy cervix and liver cells, to be compared with our HeLa and HepG2 cells). Even more interestingly, having the capability to fix cells at different times during their life cycle will likely make it possible not only to have more narrow distributions of the three characteristic parameters, but also to define characteristic values thereof for each phase of the cell life, more readily associated with the presence of hetero- and euchromatin. The present method, applied here on diffraction-limited resolution images of confocal microscopy, would also be viable with images obtained with more advanced imaging techniques such as STED or MINFLUX and benefit of the respective super-resolution.

## Supplementary Information

Below is the link to the electronic supplementary material.

Supplementary file1 (DOCX 29872 KB)

## Data Availability

The data that support the findings of this study are available from the corresponding author, upon request.
