# Supplementary material for: Image analysis tools for improved characterization of nuclear chromatin patterns by confocal fluorescence microscopy
Source: Eur Biophys J. 2025 Jun 23;55(2):241–51. doi: 10.1007/s00249-025-01770-y (PMC13109258; doi:10.1007/s00249-025-01770-y)
Supplement: Supplementary file 1 — Supplementary file1 (DOCX 29872 KB) [file 249_2025_1770_MOESM1_ESM.docx]

**Supplementary Information**


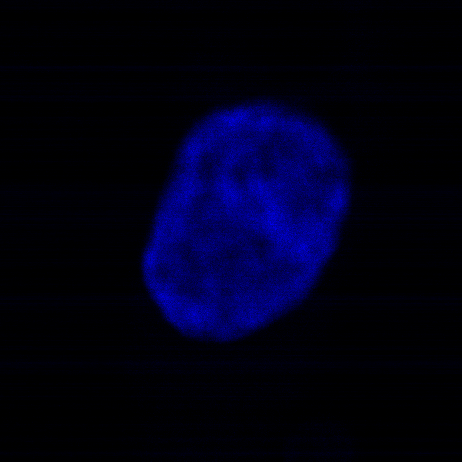

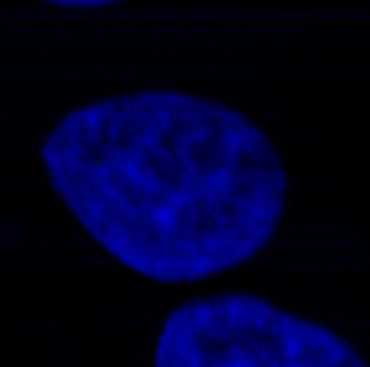

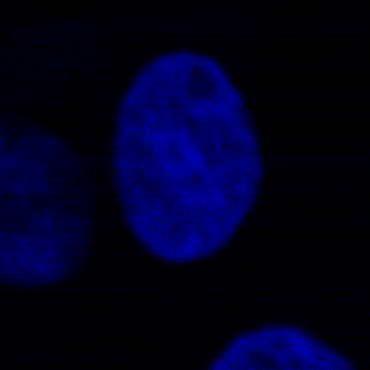

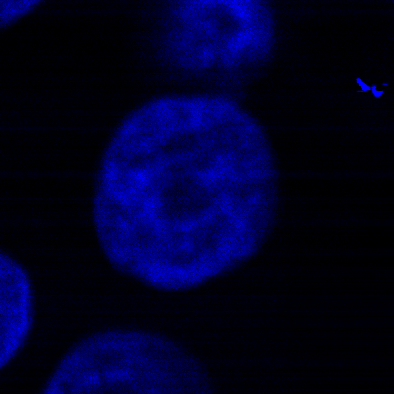

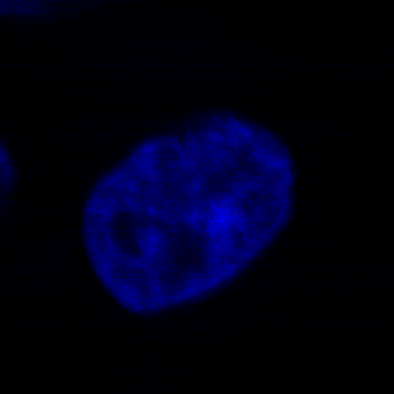

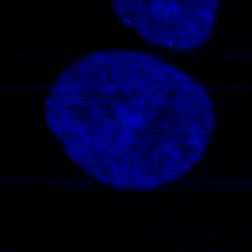

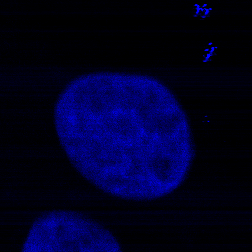

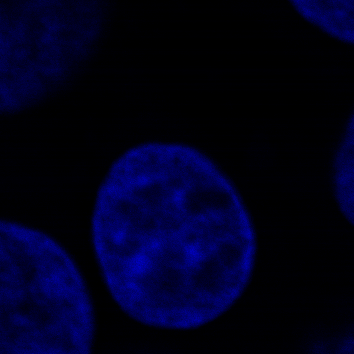

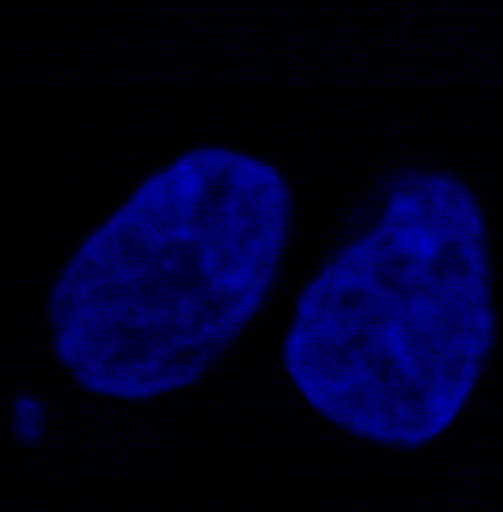

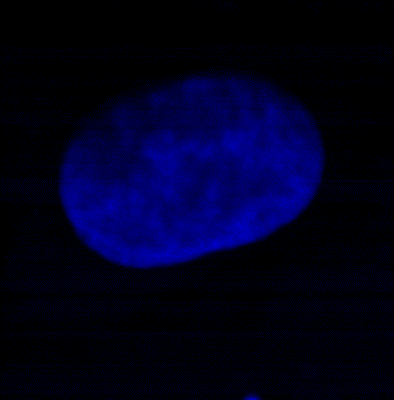

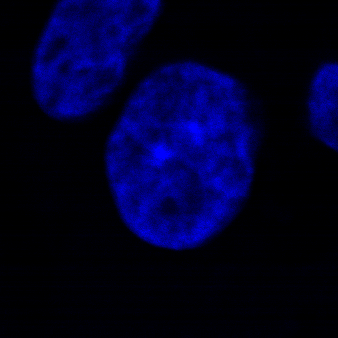

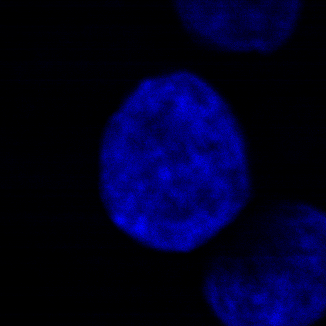

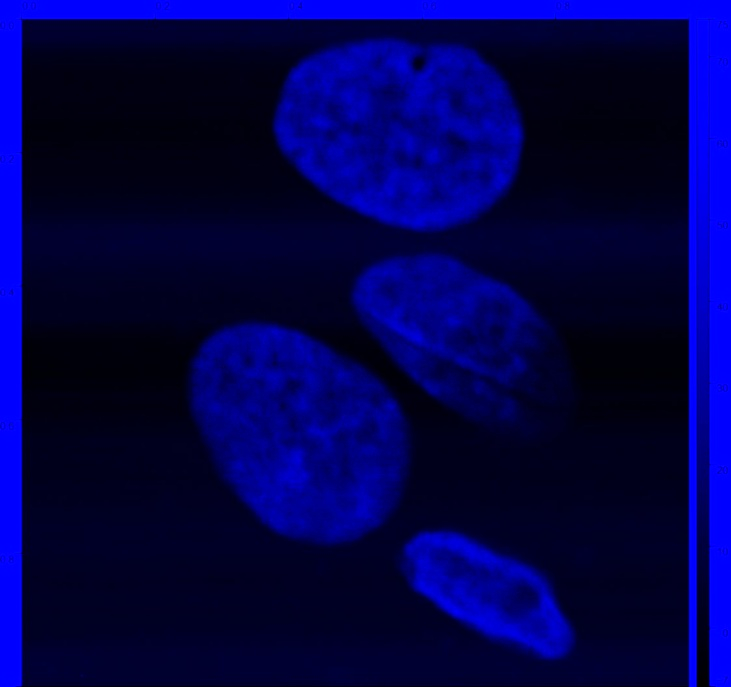

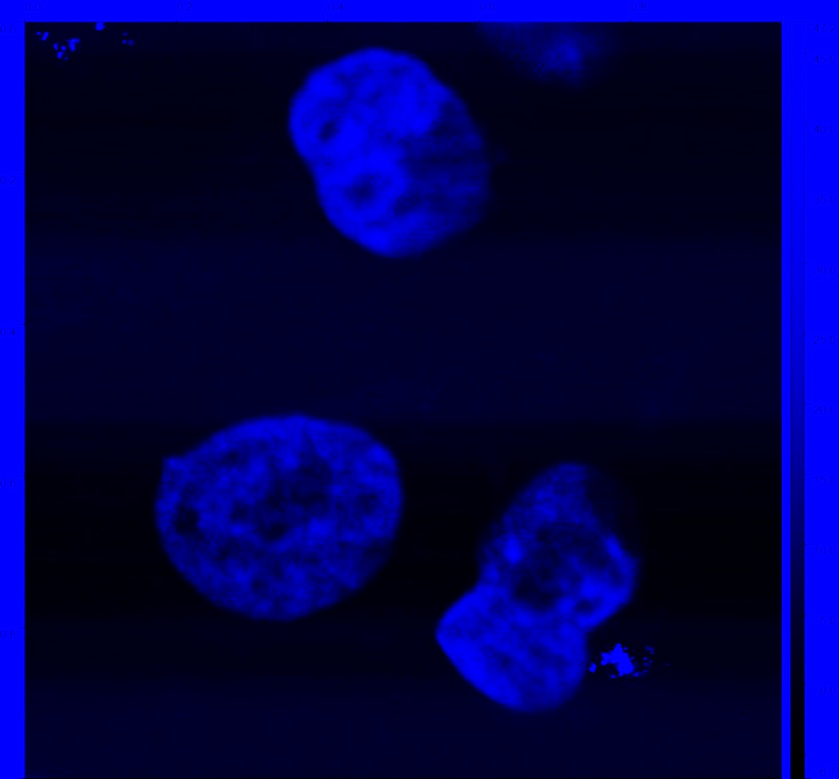

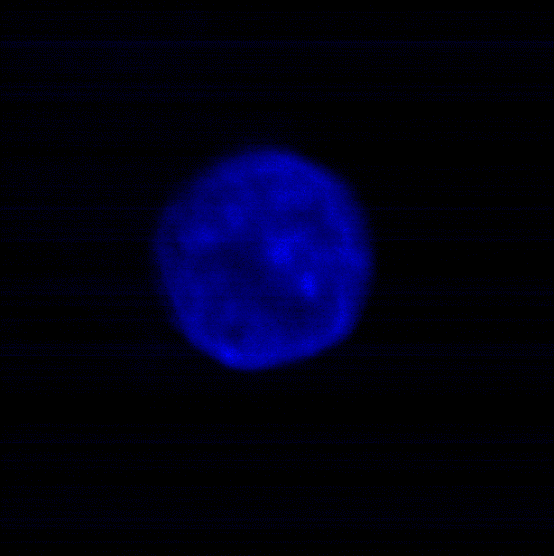

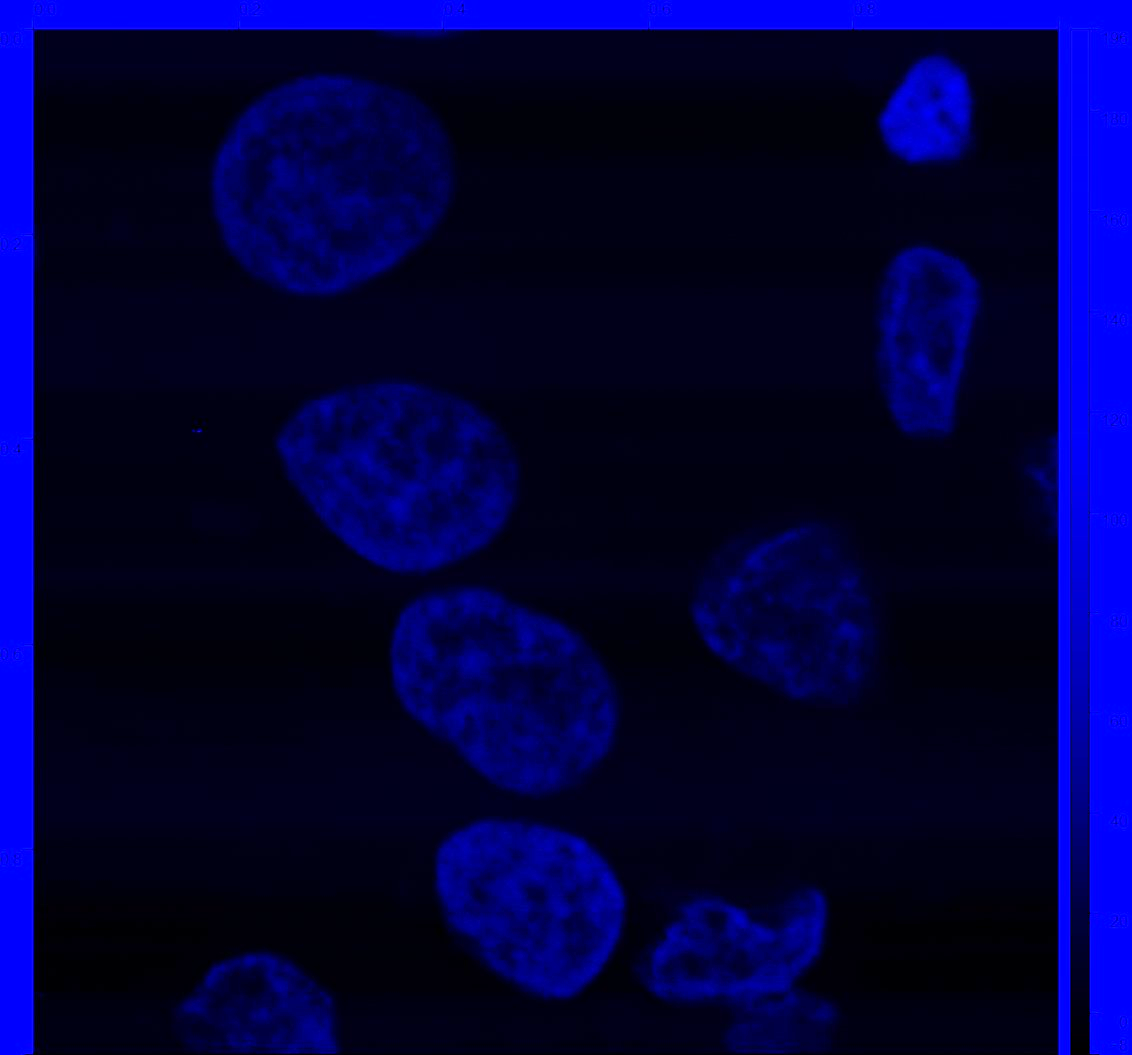

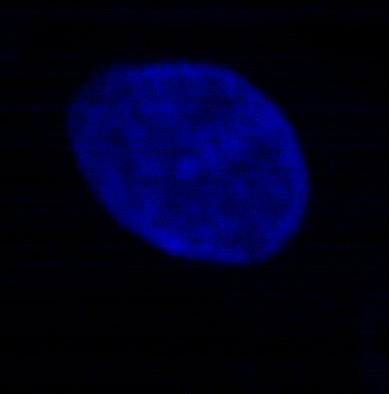

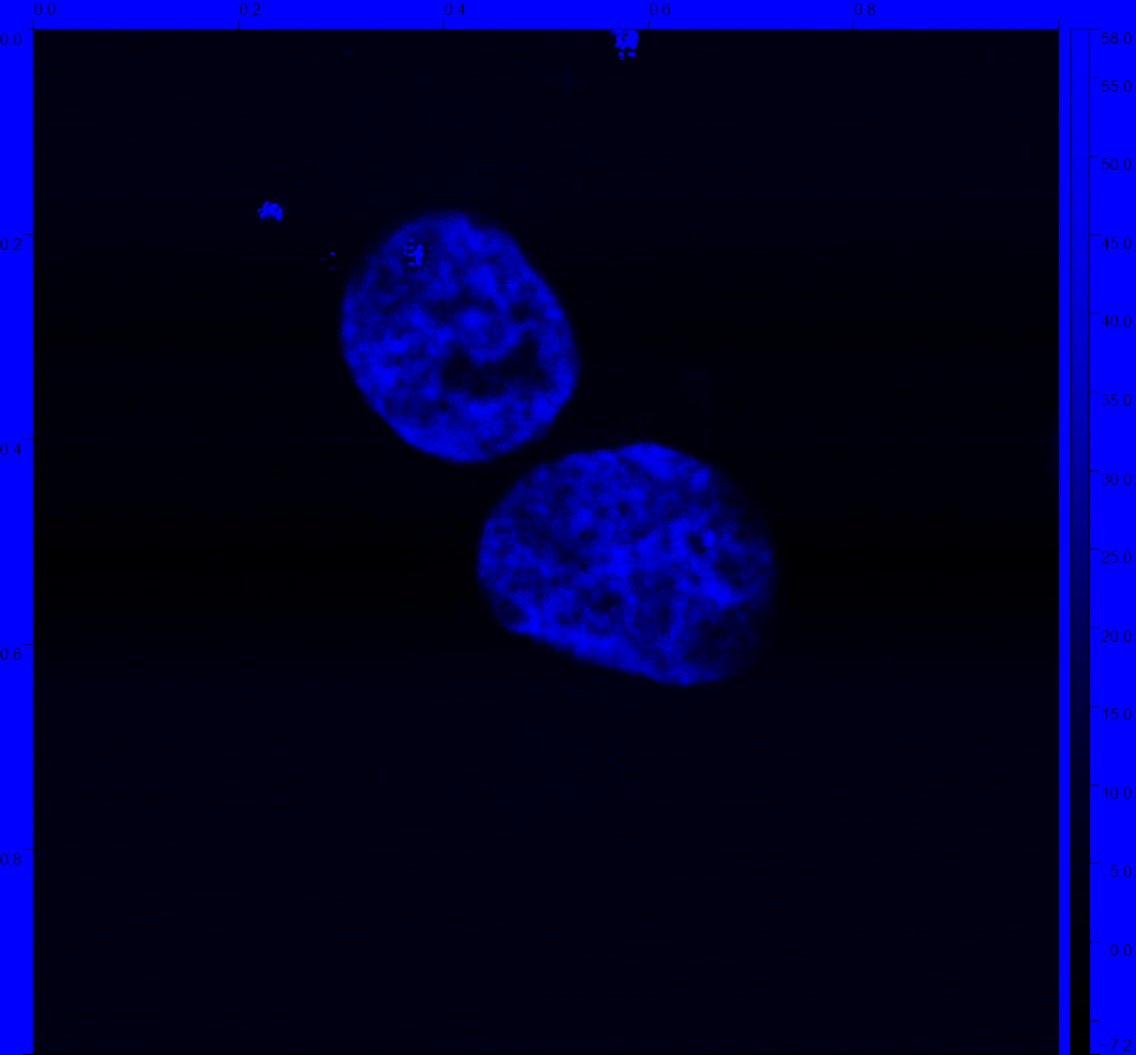

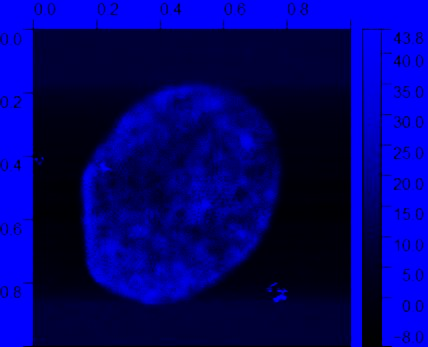

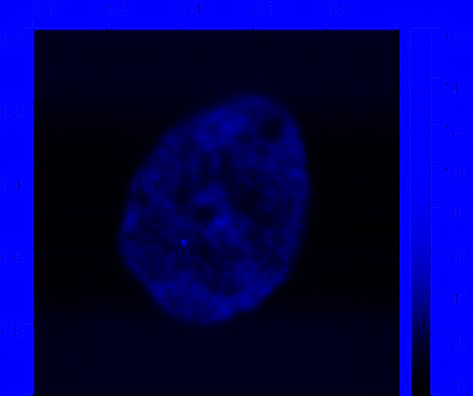


**Fig.S1**: The 20 images of HepG2 cell nuclei being part of the raw data of run#1.

Since the goal of Figs.S1,2 is to show just the image patterns for each cell type, not all the square images are the same size (each size has been optimized for visual appearance within the square window) and no single image identity within each panel has been listed (all nuclei of the same cell type are equivalent). The size range of the square image side for both Fig.sS1 and S2 was anyway between 14.4 and 28.7 μm. As a minor difference between the two populations, only a slightly higher amount of more elongated cell nuclei, similar to the case of Fig.S3d, was observed for the HeLa cells (around +15%).


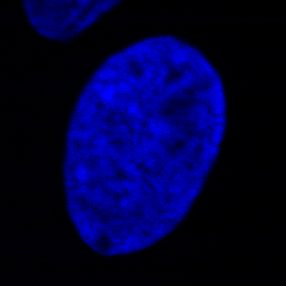

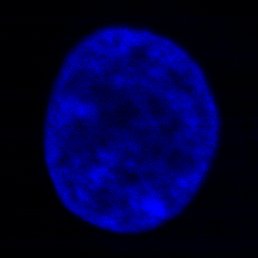

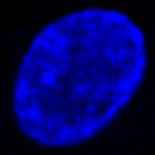

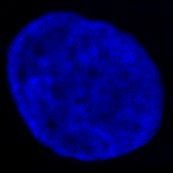

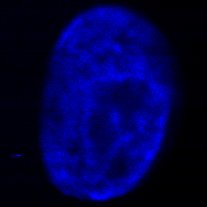

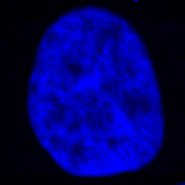

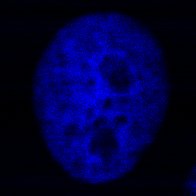

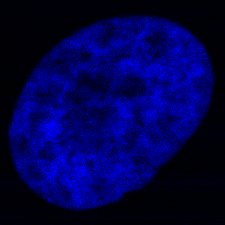

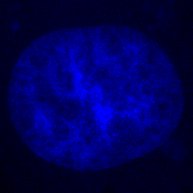

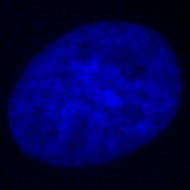

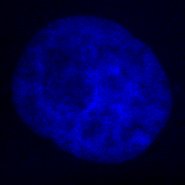

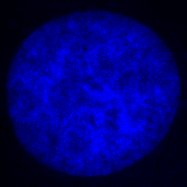

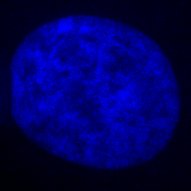

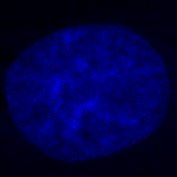

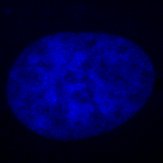

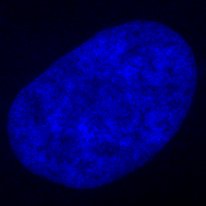

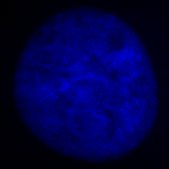

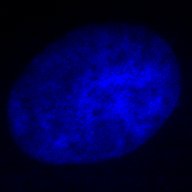

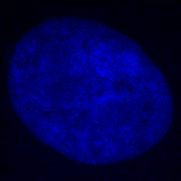

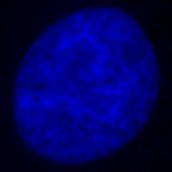


**Fig.S2**: The 20 images of HeLa cell nuclei being part of the raw data of run#1.


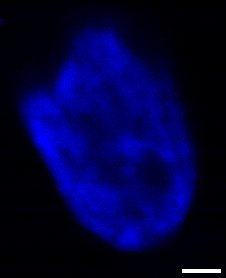


a


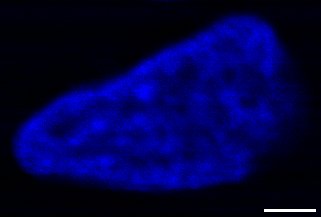


b


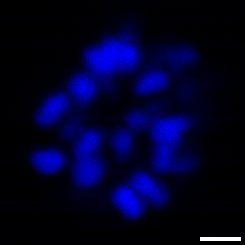


c


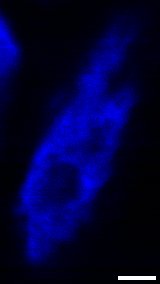


d


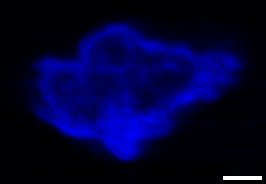


e


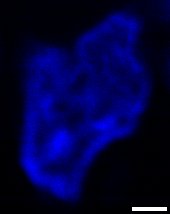


f


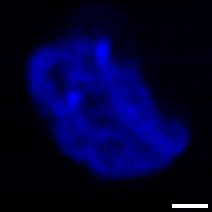


g


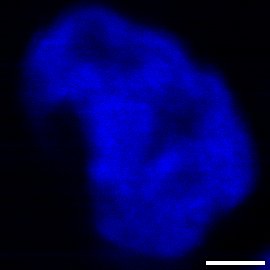


h

**Fig.S3**: Examples of images of cell nuclei that were discarded from our analysis, due to shape not fitting the case of approximately regular ellipse. a) apparently missing portion of ideal ellipse, b) triangular shape, c) apoptopic nucleus with separated residual subunits, d) excess elongated and irregular shape, e)-h) other cases of irregular shapes with protrusions and/or dents. Image aspect ratio and scale is different for each panel, however the scale bas is 2 μm for all panels.


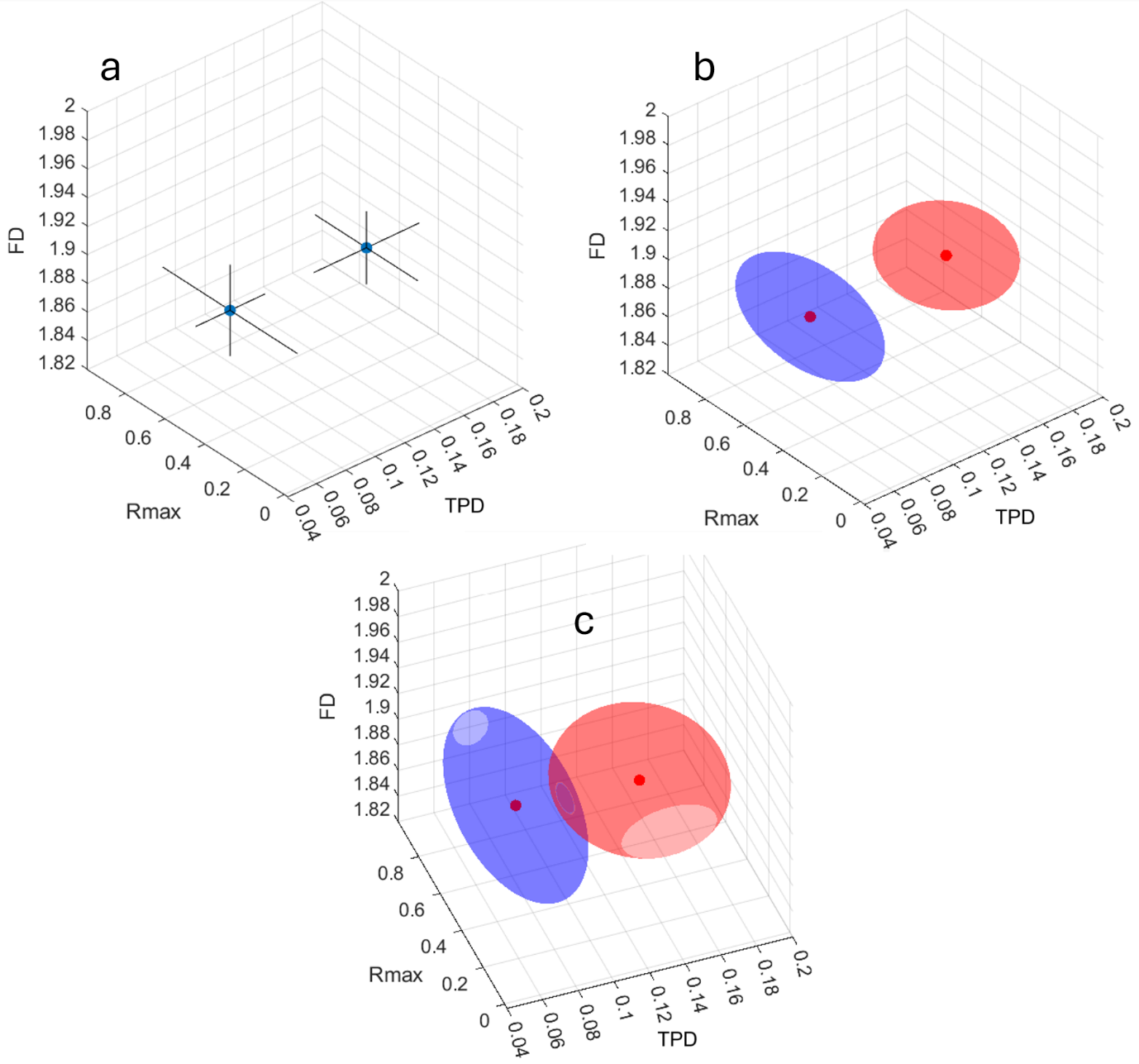


**Fig.S4:** The same data as in Fig.4c, shown via the mean datapoints only, with a statistical representation (σ is the standard deviation) of their distributions around them by: a) 3D error bars (±1 σ); b) ellipsoids showing the extension in the 3D space of the same amount (±1 σ), we see that the two populations do not touch with this figure assumed to represent their spread; c) the two ellipsoids start touching assuming a higher spread, above ±1.3 σ. Similar considerations apply to the dataset of run#2.


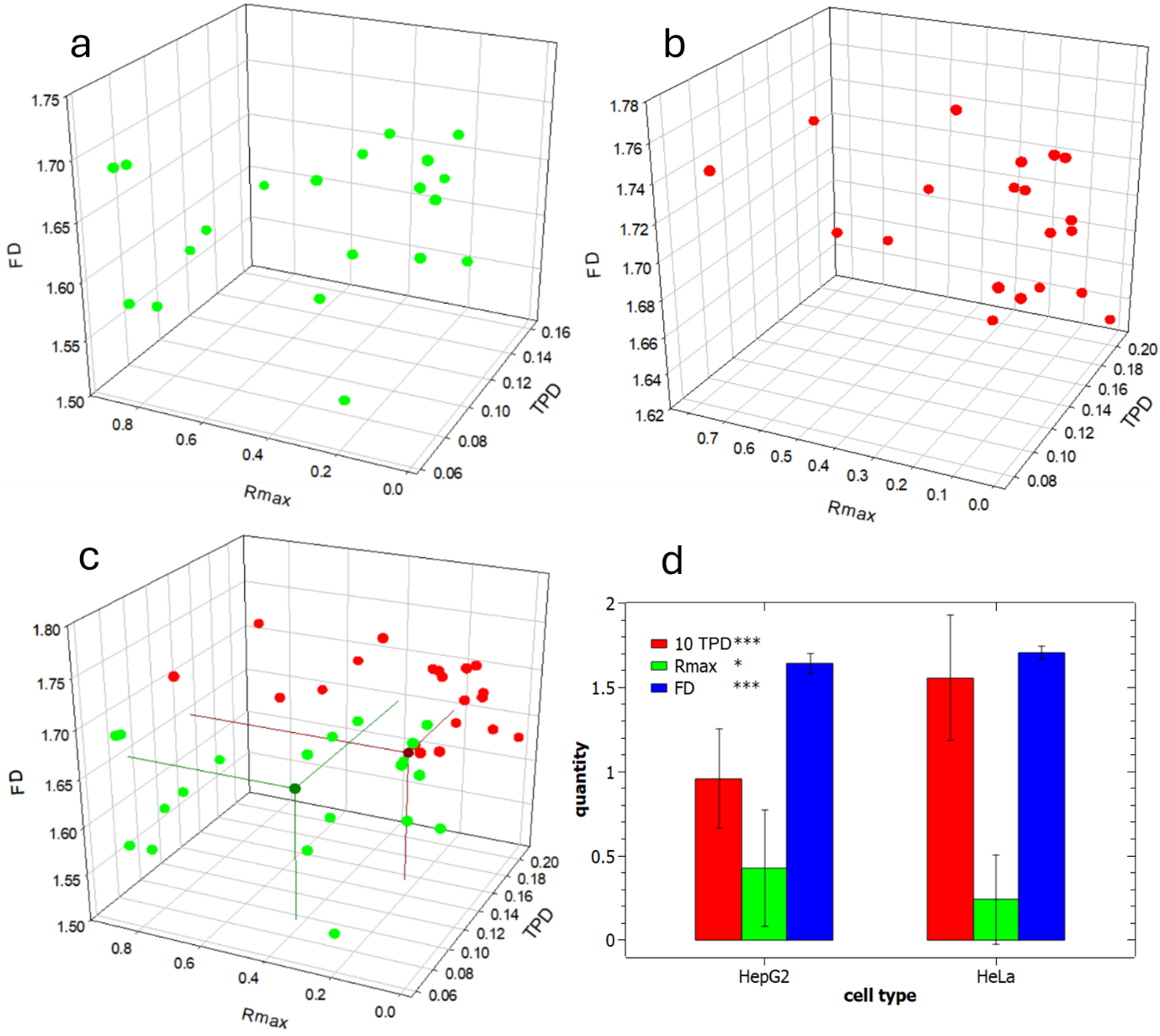


**Fig.S5:** Experimental data for comparison of HeLa and HepG2 cell nuclei chromatin patterns, during run#2 experiment. a) Datapoints for HeLa cells, b) datapoints for HepG2 cells, c) combined data of a) and b) with respective mean points, d) plot of means ± standard deviation for each of the three parameters for the two cell types; results of ANOVA showed significant difference in all cases.


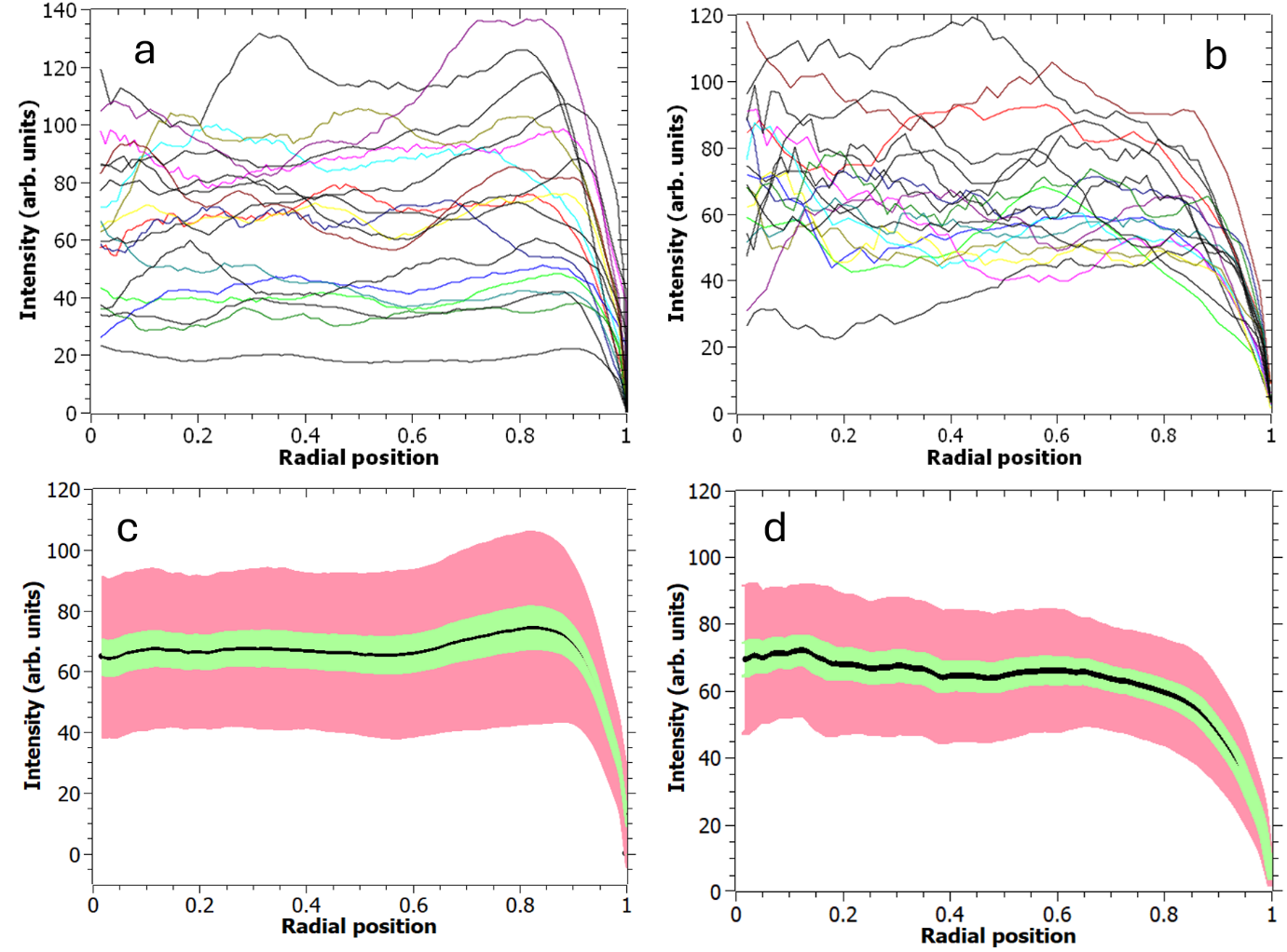


**Fig. S6:** Mean and standard deviation plots of R_max_. In addition to using all N=20 values for each nucleus type (a) HepG2, b) HeLa), here for both cell types we plot in black the resulting mean profile (c) HepG2, d) HeLa), with its uncertainty bands, with widths of either standard deviation (light red) or standard error (light green).

As the profiles obtained for each nucleus in the respective population (N=20) resulting after the processing described in Section 2.3-R_max_ had different number of datapoints, it was impossible to directly average them to obtain a single profile representing that type of nuclei. Therefore, to obtain the mean profiles in Fig.S6, before averaging them in Scidavis we first plotted treated them with command *Analysis-Interpolate*. We set 10’000 points, x range 0 to 1, and a new table of datapoints was so generated with much more closely spaced x and y values. After this step, averaging was possible with only minor error (roughly 100 pixels / 10’000 values i.e. 1%) in profile plot positions. From the columns of interpolated y values, not only the single average column was calculated, but also the standard deviation, which could be used to estimate the bands of uncertainty around the mean plot line, see Fig.S6.
